# How phosphorylation influences E1 subunit pyruvate dehydrogenase: A computational study

**DOI:** 10.1038/s41598-018-33048-z

**Published:** 2018-10-02

**Authors:** Jacopo Sgrignani, JingJing Chen, Andrea Alimonti, Andrea Cavalli

**Affiliations:** 1 Institute for Research in Biomedicine (IRB), Università della Svizzera Italiana (USI), Via Vincenzo Vela 6, CH-6500 Bellinzona, Switzerland; 2 Swiss Institute of Bioinformatics, Lausanne, Switzerland; 3 Institute of Research in Oncology (IOR), Università della Svizzera Italiana (USI), Via Vincenzo Vela 6, CH-6500 Bellinzona, Switzerland

## Abstract

Pyruvate (PYR) dehydrogenase complex (PDC) is an enzymatic system that plays a crucial role in cellular metabolism as it controls the entry of carbon into the Krebs cycle. From a structural point of view, PDC is formed by three different subunits (E1, E2 and E3) capable of catalyzing the three reaction steps necessary for the full conversion of pyruvate to acetyl-CoA. Recent investigations pointed out the crucial role of this enzyme in the replication and survival of specific cancer cell lines, renewing the interest of the scientific community. Here, we report the results of our molecular dynamics studies on the mechanism by which posttranslational modifications, in particular the phosphorylation of three serine residues (Ser-264-α, Ser-271-α, and Ser-203-α), influence the enzymatic function of the protein. Our results support the hypothesis that the phosphorylation of Ser-264-α and Ser-271-α leads to (1) a perturbation of the catalytic site structure and dynamics and, especially in the case of Ser-264-α, to (2) a reduction in the affinity of E1 for the substrate. Additionally, an analysis of the channels connecting the external environment with the catalytic site indicates that the inhibitory effect should not be due to the occlusion of the access/egress pathways to/from the active site.

## Introduction

Pyruvate (PYR) dehydrogenase complex (PDC) is a multisubunit molecular machine responsible for the conversion of PYR into acetyl-CoA through a process known as pyruvate decarboxylation^1^.

From a biochemical point of view, PDC is the gatekeeper controlling the entry of carbon into the TCA cycle (also known as Krebs cycle) from two main sources: carbohydrates and gluconeogenic amino acids.

Given its central role in cellular metabolism^2,3^, PDC has been considered a promising target for the development of antibacterial^4,5^ and anticancer drugs^6,7^. In fact, recent experimental investigations pointed out that an increase in PDC activity can sustain the progression of some cancer types (for example prostate cancer and glioblastoma)^8^ as well as the survival of cancer stem cells^7,9^.

**Figure 1:**
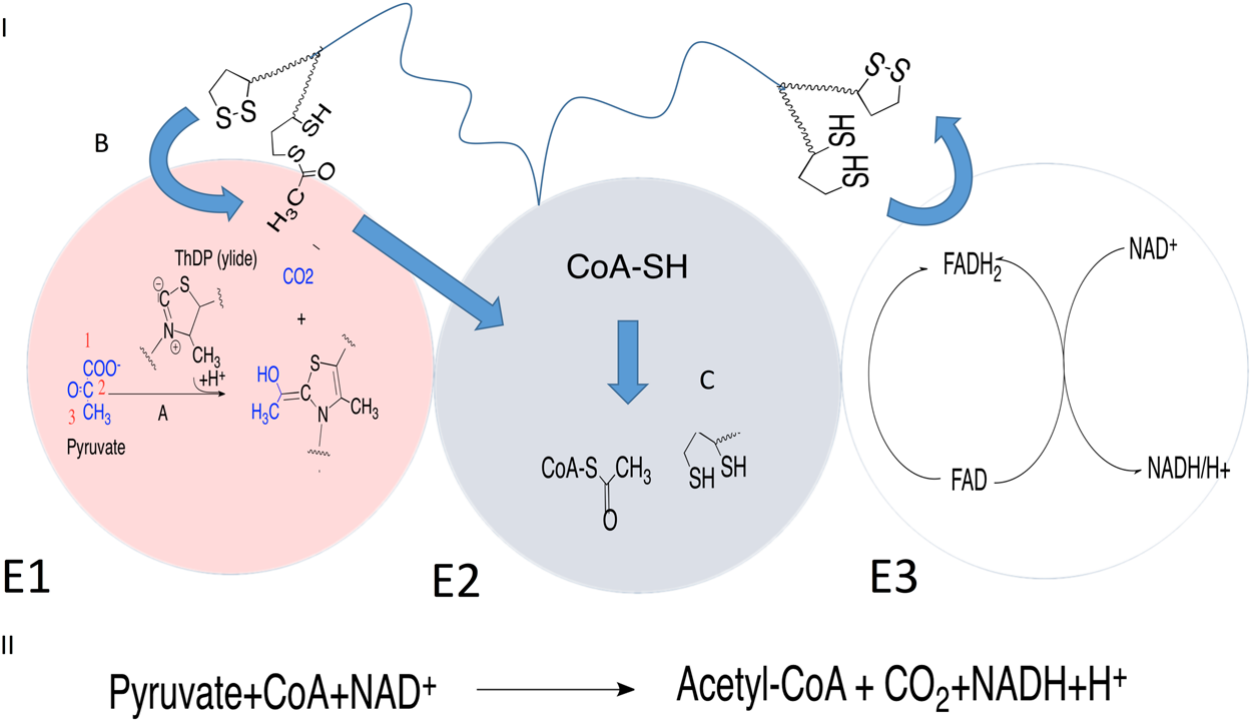
(I) Schematic view of the three PDC subunits with the corresponding reaction schemes. E1 (pink, pyruvate dehydrogenase) catalyzes pyruvate decarboxylation. E2 (blue, dihydrolipoyl trans-acetylase) catalyzes the acetyl transfer to CoA. E3 (white, dihydrolipoyl dehydrogenase) catalyzes the regeneration of the lipoamide moiety. (II) General PDC catalyzed reaction.

From the structural point of view, PDC is composed of multiple copies of three different subunits, usually called E1 (pyruvate dehydrogenase), E2 (dihydrolipoyl trans-acetylase) and E3 (dihydrolipoyl dehydrogenase)^1,10^. While the first two subunits are involved in the synthesis of acetyl-coenzyme A (Ac-CoA), the third subunit (E3) is a FAD/NAD+ dependent system performing redox recycling (Fig. 1).

Structural studies^1^ have shown that the E1 PDC subunit is formed by two different chains (α and β) and is characterized by the presence of a magnesium ion coordinated by three amino acid residues (Asp167-α, Αsn196-α, Τyr198-α) and a thiamine diphosphate (ThDP) molecule acting as a cofactor. ThDP is directly involved in the enzymatic reaction where after activation by Glu59 and the consequent formation of a dipolar molecule bearing a formally negatively charged atom (Fig. 1)^1,11^ called ylide, it carries out a nucleophile attack on the PYR molecule to form a covalent ThDP-acetyl adduct (step A, Fig. 1). Then, the reaction proceeds with the involvement of the E2 subunit. This second enzymatic subunit bears a long amino acidic arm ending with a lipoamide group, which is able to swing between the E1 and E3 active sites. In particular, the acetyl group from the ThDP-acetyl in the E1 subunit is first transferred to the lipoamide. Then, the swinging arm moves out of the E1 subunit and enters into the E2 catalytic site, where the acetyl group is transferred to the CoA molecule to form the Ac-CoA. Finally, the arm moves to the E3 catalytic site, where the initial oxidation state of the lipoamide group is restored.

Given its importance in cellular metabolism, the PDC complex has been at the center of numerous structural and functional studies^1^. However, several questions regarding (1) the PDC global stoichiometry, (2) the influence of posttranslational modifications on the function of PDC and (3) a full characterization of the enzymatic reaction at an atomistic level are still unanswered.

In regards to question (2), it has been extensively reported that the activity of PDC is regulated by the phosphorylation of three serine residues^1^ and that the level of phosphorylation is controlled by four pyruvate dehydrogenase kinases (PDKs1-4) and two pyruvate dehydrogenase phosphatases (PDP1-2).

In particular, experimental data clearly indicate that the phosphorylation of specific serine residues (Ser-264-α, Ser-271-α, and Ser-203-α in the human sequence, subsequently indicated as Ser-264-α-P, Ser-271-α-P and Ser-203-α-P when phosphorylated) results in different degrees of enzyme inhibition, with the strongest effect observed with Ser-264-α-P^1,6^.

*In vitro* studies demonstrated^12^ that PDK1 is the only kinase that can phosphorylate all the three sites, even if with different preferences and kinetics, while PDK2, PDK3 and PDK4 only act on Ser-264-α and Ser-271-α. In contrast, a similar action on all the three sites was observed for the two phosphatases^13^.

Furthermore, other investigations have shown that these posttranslational modifications are independent and not sequential^13,14^ and that the phosphorylation of Ser-264-α drastically reduces the enzymatic activity, while the phosphorylation of Ser-271-α or Ser-203-α have less clear inhibitory effects^13,15,16^.

Finally, experimental investigations^6,17,18^ indicated that the levels of PDC phosphorylation are significant for several widely diffused diseases such as cancer^6,17,18^ diabetes and neuro-metabolic disorders^19,20^. Understanding, at an atomistic level of detail, how posttranslational modifications alter enzymatic activity could open the way to the development of innovative therapeutic tools based on PDC inhibition/activation.

Two different hypotheses to explain the PDC inhibition induced by phosphorylation have been proposed in the scientific literature^21,22^. Kato *et al*.^21^ suggested that a perturbation of protein dynamics, in particular of two phosphorylation loops (loop A: from 259-α to 282-α, loop B: from 195-α to 205-α), as origin of the inhibition while Seifert *et al*.^22^ hypothesized that Ser-264-α-P could hinder the substrates access to the active site^22^.

Over the years, molecular simulations have become a valuable tool to answer these types of questions^23–34^ as they provide a detailed atomistic picture of the biomolecular dynamics. In particular, they have been recently used to obtain a low resolution model of the entire PCD structure^35^ and to investigate other aspects of this enzymatic complex^36,37^.

Thus, we carried out an MD simulation study in which the PDC-E1 system in its unphosphorylated and phosphorylated forms (Ser-264-α-P, Ser-271-α-P and Ser-203-α-P) was simulated for 1 μsec (MD). Finally, the trajectories were analyzed to investigate the effects of the posttranslational modifications on the protein structure, dynamics and on its ability to bind the substrate.

## Results and Discussion

### Analysis of molecular dynamics simulations

The molecular dynamics trajectories for the different systems were compared by considering different quantities such as RMSD, RMSF, Lindemann coefficient and pyruvate binding energy, as well as, the number, position, length and diameter of the tunnels leading to the active site.

**Figure 2:**
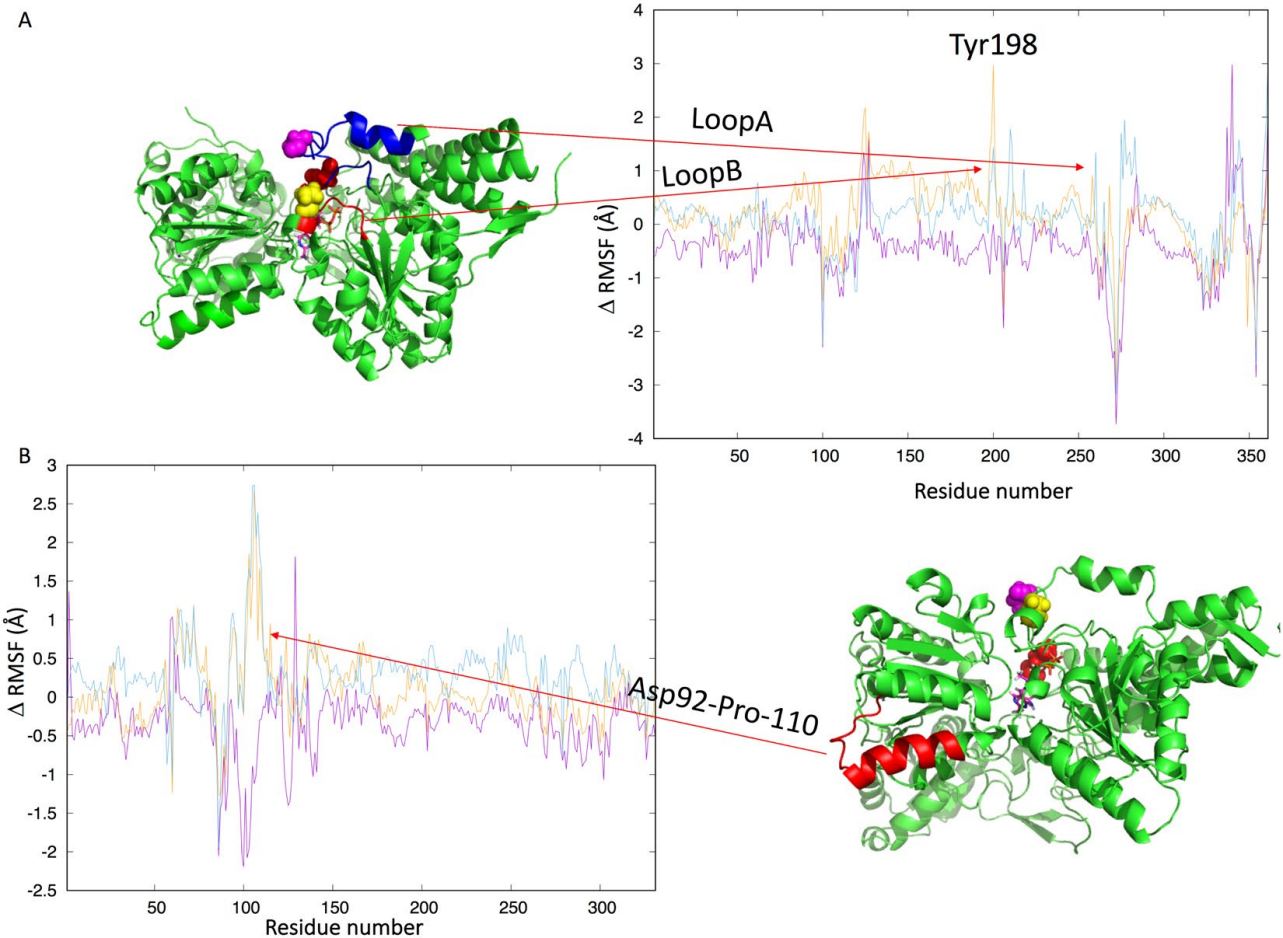
Differences between the RMSF values measured for the three phosphorylated species, Ser-264-α-P (light blue), Ser-271-α-P (orange), Ser-203-α-P (violet), and the WT. Positive values correspond to an increment of RMSF values with respect to WT, while negative values correspond to a reduction. The data for the α (**A**) and β (**B**) subunits are reported in separate diagrams for clarity.

**Figure 3:**
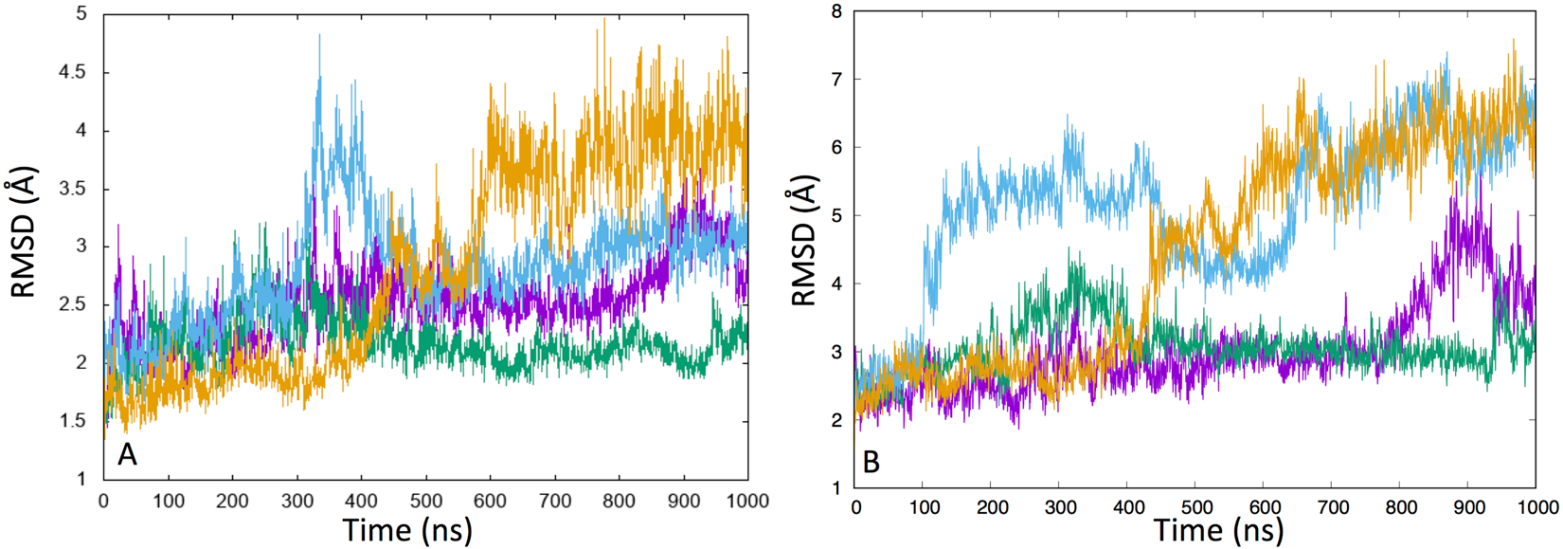
(**A**) Backbone RMSD calculated with respect to the starting coordinates of the WT protein over the entire MD simulations. WT (violet) and the three phosphorylated species, Ser-264-α-P (light blue), Ser-271-α-P (orange), and Ser-203-α-P (green)). (**B**) Binding site RMSD (His63-α, Met200-α, His263-α, Phe61-α, Tyr89-α, Met82-β) calculated for the entire residues (backbone + sidechains) over the entire simulation time. WT (violet) and the three phosphorylated species Ser-264-α-P (light blue), Ser-271-α-P (orange), and Ser-203-α-P (green).

**Figure 4:**
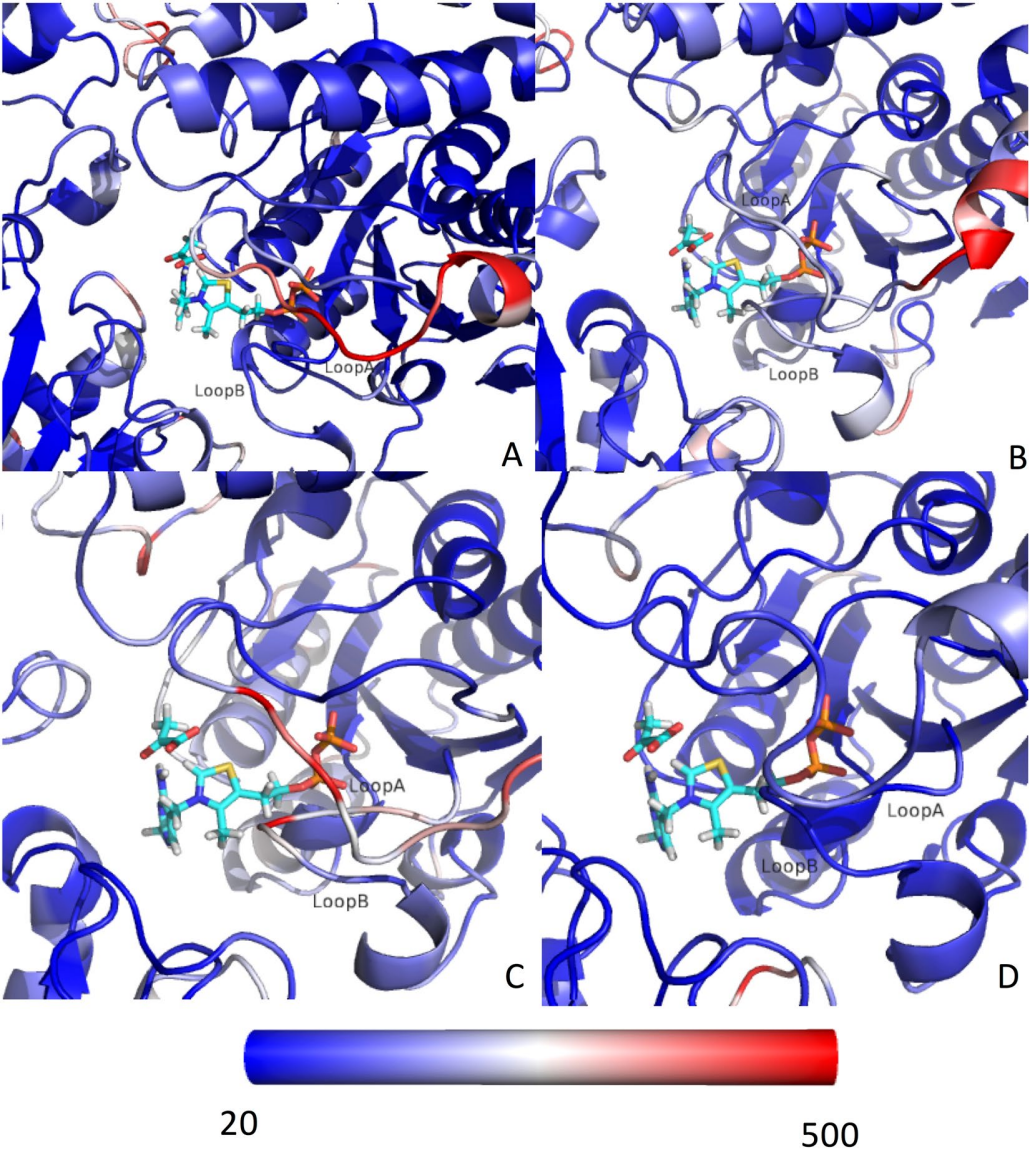
PDC-E1 structure colored by calculated B-factor (computed with the RMSF tool implemented in GROMACS 5.1.4). (**A**) Wild type, (**B**) Ser-264-α-P, (**C**) Ser-271-α-P, (**D**) Ser-203-α-P.

A detailed analysis of our simulations (Figs 2–4 and Table 1) clearly shows that Ser-264-α-P and Ser-271-α-P have a different influence on the protein structure and dynamics when compared to Ser-203-α-P. In particular, RMSD and RMSF analyses of the protein backbone carried out on the entire 1 μs MD simulations showed that Ser-203-α-P stabilizes the protein in a conformation that is more similar to that observed in X-ray experiments (PDB ID: 1NI4) and reduces atomic fluctuations (lower RMSF). In contrast, higher RMSD and RMSF values were observed for Ser-264-α-P and Ser-271-α-P.

**Table 1:** Lindemann coefficients calculated for the entire protein, loop A and loop B over the four 1 µs MD simulations.

	WT	Ser-264-α-P	Ser-271-α-P	Ser-203-α-P
Entire protein	0.27	0.29	0.28	0.23
Loop A	0.36	0.35	0.32	0.27
Loop B	0.27	0.31	0.33	0.23
Asp92-β to Pro-110-β	0.32	0.39	0.38	0.23

Next, we aimed to quantify the observed differences in protein dynamics by computing the Lindemann coefficient^38^, which gives an estimation of the solid-liquid behavior of a protein. The values of the coefficients (Table 1) computed for the entire protein as well as for different portions, confirm a stiffening of the entire molecule induced by Ser-203-α-P. Moreover, the values indicate that phosphorylation of Ser-264-α and Ser-271-α increases the flexibility of the region from Asp-92-β to Pro-110β, while that on Ser-203-α rigidifies the same region.

A different trend was observed for the two phosphorylation loops (for loop definitions, see introduction). In fact, while Ser-203-α-P stiffens both loops, Ser-264-α-P and Ser-271-α-P significantly increase the flexibility of loop B but not that of loop A. Concerning this point, it should be noted that Ser-264-α and Ser-271-α are inside loop A, while Ser-203-α is placed in loop B.

To better understand the origins of these differences, we visually inspected the trajectories of all the simulations.

This analysis showed that during the Ser-203-α-P simulation, this residue gets surrounded by three arginine residues (Arg273-α, Arg206-α, Αrg197-α) forming a cationic-hole (Fig. SI1). Interestingly, Arg273-α is located in the terminal region of loop A; thus, the interaction with this residue could reasonably be at the origin of the greater rigidity of this loop during the MD simulations with the phosphorylated Ser-203-α. Α similar, but less stable, cationic hole is formed around Ser-271-α-P that interacts with Lys39−β, Arg273-α and Arg275-α (Fig. SI2). In contrast to Ser-271-α-P and Ser-203-α-P, similar interactions were not visible for Ser-264-α.

Several experimental and theoretical investigations^21,39,40^ have pointed out the role of specific amino acids residues in the catalytic activity of E1-PCD. We, therefore, analyzed our simulations focusing on the consequences of phosphorylation on the catalytic site structure and dynamics.

The RMSD values (Fig. 3B), computed taking into account all atoms of the amino acids residues forming the catalytic site (His63-α, Met200-α, His263-α, Phe61-α, Tyr89-α, Met82-β), showed that Ser-264-α-P and Ser-271-α-P perturb the starting structure more significantly than Ser-203-α-P.

In summary, the analysis of our simulations shows that phosphorylation of the three investigated serine residues have marked structural and dynamics effects on the PDC-E1-α subunit. In particular, both Ser-264-α-P and Ser-271-α-P increase the flexibility of loop B, the loop that directly coordinates the magnesium ion interacting with the ThDP cofactor. These findings are in line with the conclusions of Kato *et al*.^21^ that also pointed out an increased disorder in the two phosphorylation loops (loop A and B).

At the same time, the stiffening of the protein structure observed in the presence of Ser-203-α-P, suggests that this posttranslational modification should not inhibit PDC or, alternatively, that the inhibition could have a different explanation.

Phosphorylation of single residues can influence protein properties such as hydrophobicity, lipophilicity^41^ and conformational entropy^42^. We, therefore, used specific computational tools to analyze our simulations with respect to these properties.

The entropic contribution (conformational entropy) to the total free energy (−ΤΔS) was estimated to be −3.4 ± 0.1, −5.5 ± 0.1 and 26.2 ± 0.1 kcal/mol for the systems bearing Ser-264-α-P, Ser-271-α-P and Ser-203-α-P, respectively. These values confirm a stabilizing effect of Ser-203-α-P and a slight destabilizing action of Ser-264-α-P and Ser-271-α-P.

Following the suggestion of Polyansky and Zagrovic^41^, we also investigated the effects of phosphorylation on the protein hydrophobicity/lipophilicity by computing the molecular hydrophobicity potential (MHP) on multiple snapshots extracted from our MD simulations.

These calculations resulted in ΔMHP values of −994, −835 and 258 log P units for the systems with Ser-264-α-P, Ser-271-α-P and Ser-203-α-P, respectively. These results indicate a decrease in the hydrophobicity of the protein in the first two cases and an opposite effect in the last one.

### Influence of phosphorylation on pyruvate binding affinity

Our previous calculations, as well as experimental studies^14^, suggest that changes in the enzyme affinity for the substrate and/or the cofactors could be one of the consequences of the phosphorylation. Because of technical problems in the purification of phosphorylated proteins, experimental investigations have been carried out using glutamate and aspartate mutations of the serine amino acids to mimic the effects of phosphorylation. However, while these mutations effectively reproduce the negative net charge of the phosphate group, they less efficiently mimic other properties, in particular the amino acid size. Therefore, to better clarify this point, we estimated the affinity between PDC-E1 and PYR or ThDP in all the investigated systems by MM-GBSA (Table 2).

**Table 2:** Binding energy estimated for PYR and ThDP by MM-GBSA.

	WT	Ser-264-α-P	Ser-271-α-P	Ser-203-α-P
ΔG_PYR_	−1 (0.05)	5.3 (0.06)	−1.6 (0.1)	−4.4(0.06)
ΔG_ThDP_	−103.1 (0.5)	−116.2 (0.4)	−103.2 (0.5)	−113.6 (0.4)

The reported values are expressed in kcal/mol, and the standard error of the mean is reported in parenthesis.

**Figure 5:**
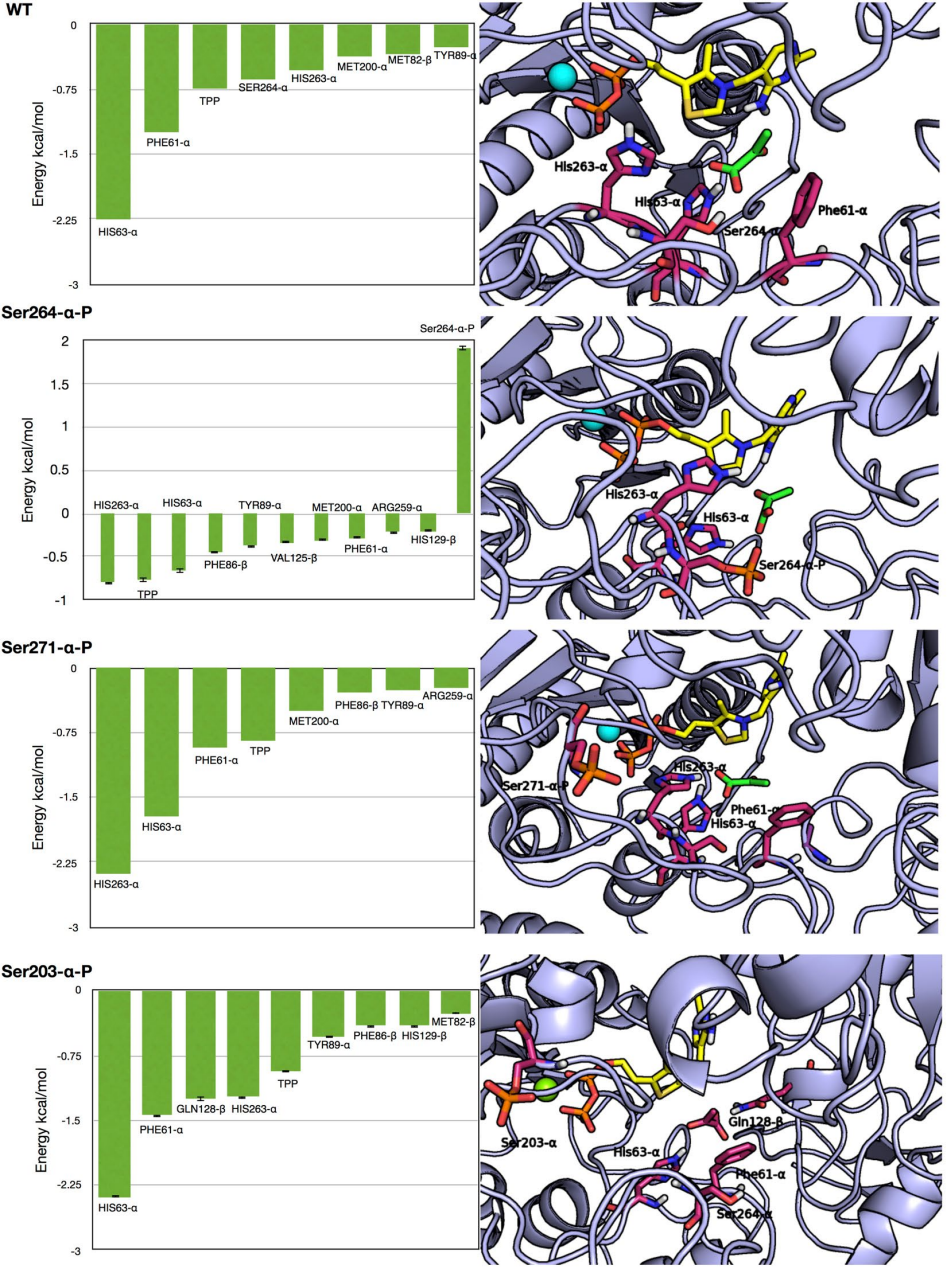
(Left column), the results of per-residue Energy decomposition carried out for the WT (**A**) and the systems bearing a Ser-264-α-P, Ser-271-α-P, or Ser-203-α-P. For sake of clarity, only contributions higher the 0.2 kcal/mol (absolute value) were reported. (Right column) representative structure of the catalytic site in the four investigated systems. The residues with the higher contributions to the binding and the phosphorylated residues are depicted as sticks; the nonpolar hydrogens are not shown for the sake of clarity.

For PYR, our calculations indicate that Ser-264-α-P significantly reduces the enzyme affinity. In contrast, Ser-203-α-P increases the affinity for PYR, while Ser-271-α-P has no significant effect. Next, we performed a per-residue decomposition of the binding energy to better understand which residues facilitate or antagonize the binding of the substrate (Fig. 5).

From the analysis of these results, we noticed that the network of residues with the strongest contribution to the binding energy is generally conserved among all the investigated systems. However, in the case of the Ser-264-α-P mutant, we observed a large (~2 kcal/mol) positive, i.e. repulsive, contribution from Ser-264-α-P and a reduction (~1.5 kcal/mol with respect to the unphosphorylated protein) in the contribution to the binding energy given by His63-α and His263-α. In fact, in the WT protein, these two residues are close to each other on the interior of the active site and are able to form stable H-bond interactions with PYR. In the system with Ser-264-α-P, the phosphate group interacts with His263-α, inducing its reorientation to the exterior of the active site and resulting in the breaking of the H-bonds with the substrate.

In the case of Ser-203-α-P, the increase in the predicted affinity, with respect to the unphosphorylated protein, is due to a larger contribution of Phe61-α and an additional interaction with Gln128-β.

The calculations carried out to estimate the protein affinity for ThDP did not show any negative effects of the phosphorylation of the three serine residues. In contrast, the computed affinities for the proteins bearing Ser-264-α-P and Ser-203-α-P were higher than for the unphosphorylated ones.

In summary, MM-GBSA calculations indicated that Ser-264-α-P has a repulsive effect on PYR reducing its affinity for the catalytic site, while a similar effect was not observed for Ser-271-α-P and Ser-203-α-P. Moreover, the results indicated that serine phosphorylations do not affect the affinity for ThDP.

### Analysis of the protein channels by Caver3.0

Seifert *et al*.^22^, studying the structure of the a pseudophosphorylated variant in which Ser-264-α was mutated to glutamate, concluded that the Ser-264-α-P hinders the substrate access to the active site. However, their conclusions were drawn by structural investigation on a system bearing a phosphomimetic mutation and using an experimental technique, X-ray crystallography, that provides only limited information about the dynamics of the system. Furthermore, the study^22^ focused on only one of the three possible phosphorylations.

Therefore, aiming to determine if this hypothesis could be confirmed by simulations of the different systems bearing an explicit phosphate group, we analyzed the 1 μs MD simulations outputs with Caver3.0^43^, a software specifically designed to identify tunnels in protein and previously used in similar investigations^25,44,45^.

**Figure 6:**
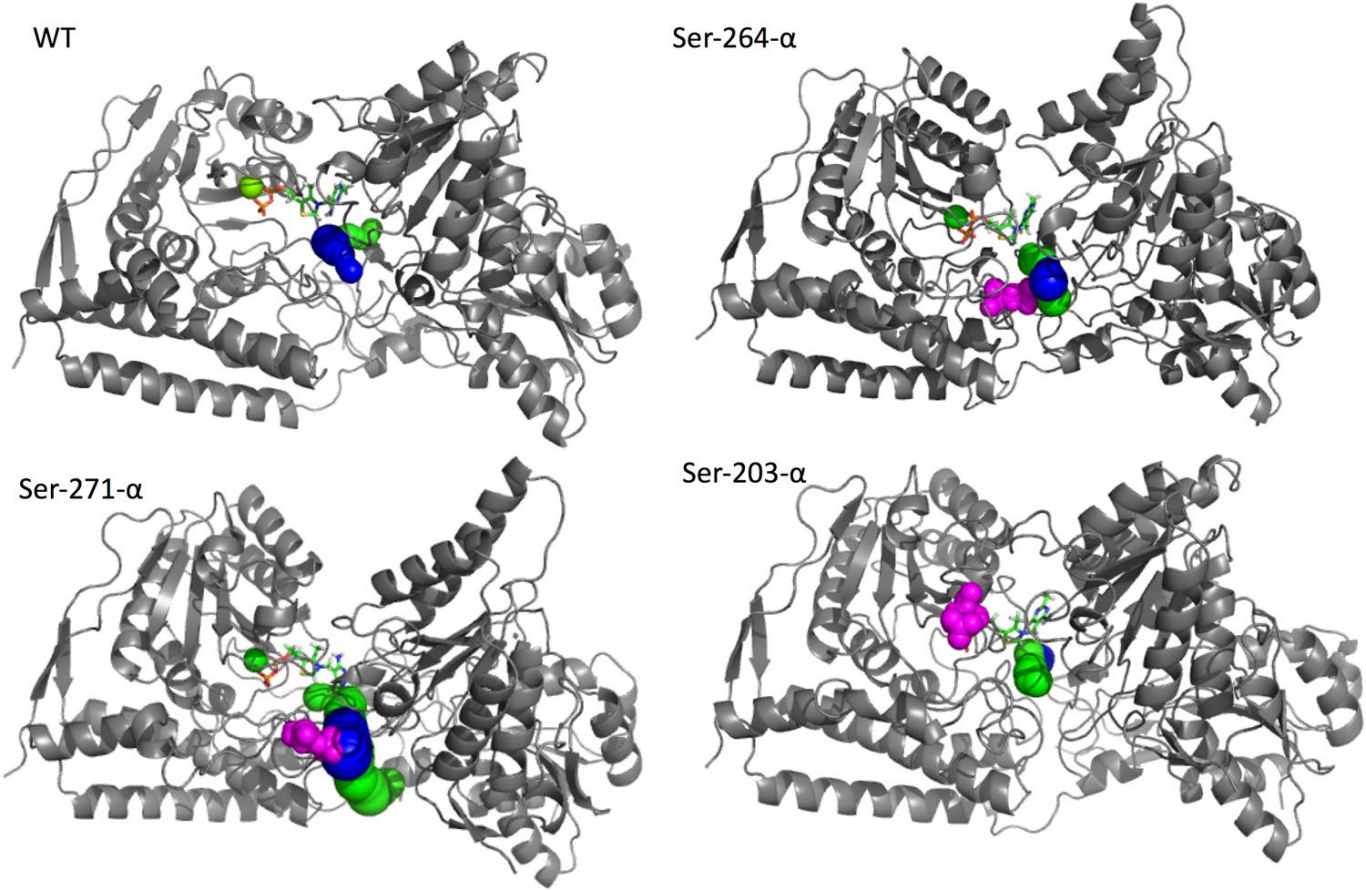
Graphical representation of the Caver3.0 results. The first and the second tunnels are represented in blue and green, respectively. The three phosphorylated serine residues are always depicted in magenta as spatial references. In the cases of Ser-264-α-P and Ser-271-α-P, in which the tunnels have a common pathway, only one color is used to depict the common part.

In the case of the WT simulation, the tunnel with the best score identified in the 65% of the analyzed snapshots and corresponding to that described by Tittmann and coworkers^22^ is topologically located near the protein region occupied by Ser-264-α. Moreover, a second channel (present in the 20% of the snapshots, Fig. 6 and Table 3) is detected on the other site of the protein. For both tunnels, the average bottleneck diameter is larger than 3.0 Å, which is enough to allow the transit of a PYR molecule.

**Table 3:** Summary of the Caver3.0 calculations.

Tunnel Number	Total snapshots	Average bottleneck radius (Å)	Maximum bottleneck radius (Å)	Average length (Å)
WT				
1	815/1250	1.7 ± 0.2	2.5	9 ± 3.5
2	271/1250	1.6 ± 0.1	2.0	8.3 ± 2.9
3	131/1250	1.6 ± 0.1	1.9	12.3 ± 3.1
4	107/1250	1.6 ± 0.1	2.0	11.1 ± 3.3
Ser-271-α64				
1	927/1250	1.9 ± 0.2	2.8	15 ± 7.3
2	943/1250	1.8 ± 0.2	2.9	16.1 ± 4.5
3	533/1250	1.8 ± 0.2	2.7	16.3 ± 4.2
4	401/1250	1.6 ± 0.1	2.3	35.2 ± 7.8
Ser-271-α71				
1	860/1250	2.0 ± 0.3	3.2	14.7 ± 4.8
2	599/1250	2.0 ± 0.3	3.0	23.6 ± 6.5
3	347/1250	1.8 ± 0.2	2.6	16.9 ± 6.1
4	373/1250	1.8 ± 0.4	2.5	24.6 ± 4.5
14				
1	848/1250	1.8 ± 0.2	2.6	10 ± 4.1
2	758/1250	1.6 ± 0.1	2.5	11 ± 3.5
3	330/1250	1.6 ± 0.1	2.5	7.5 ± 2.7
4	51/1250	1.6 ± 0.1	1.8	25.5 ± 4.1

The tunnels are ranked by priority score, a specific tunnel scoring function implemented in Caver3.0^44^.

In addition, in the case of the Ser-264-α-P system, the best scoring channel still corresponds to that which was proposed from the observation of the X-ray structure and passes near Ser-264-α-P^22^. This channel is characterized by an average bottleneck radius larger than that measured for the same channel in the WT and it is present in the 74% of the analyzed snapshots. A second channel, starting from a slightly different position but converging in tunnel 1, was also detected in the 75% of the snapshots.

A similar behavior was observed for Ser-271-α-P. In fact, in this case, the first two channels (detected in the 69% and 48% of the snapshots) start from different positions but in their terminal portion, converge on the same pathway passing near the regions of the protein occupied by Ser-264-α and Ser-271-α. It is also worth noting that in these cases, the channel diameters are larger than those detected for the main channels of the WT system.

Finally, in the case of Ser-203-α-P, the two best scoring channels detected by Caver were essentially identical to those observed in the WT, only with a reverse ranking.

Taken together, the results of our analysis strongly suggest that the channel passing near Ser-264-α and Ser-271-α, corresponding to what is already described in the literature^22^, should represent the main pathway connecting the protein surface with the active site. In all the phosphorylated systems, this channel was demonstrated to be as viable as in the WT system. We can therefore conclude that the origin of the inhibitory effect of phosphorylations^15,16,21^ is not due to the closure of the access route to the active site.

## Summary and Conclusions

In this work, we focused on the effects of specific serine phosphorylations on the enzymatic activity of the PDC E1 subunit.

Our calculations highlighted an increase in the protein flexibility induced by the phosphorylation of Ser-264-α and Ser-271-α at both the global (entire protein) and local (catalytic site) levels, while the phosphorylation of Ser-203-α was associated with an increase in protein rigidity. In particular, in the Ser-264-α-P and Ser-271-α-P systems, we observed a significant increase in the flexibility of the phosphorylation loop B, which is directly involved in the binding of both the ThDP cofactor and of the ThDP coordinated Mg2^+^ ion.

Binding energy calculations helped to shed light on the consequences of the increased flexibility induced by the presence Ser-264-α-P and Ser-271-α-P. In fact, while the affinity for the ThDP cofactor is unaltered, Ser-264-α-P reduces the affinity of PYR for the catalytic site. A similar effect was not observed for Ser-271-α-P, while the affinity in the Ser-203-α-P system was significantly increased by the posttranslational modification.

Finally, Caver calculations showed that the presence of the phosphate groups in the three considered serine residues does not induce a closure of the access/release pathway to/from the catalytic site.

Taken together, our findings suggest that the strong inhibitory effect observed for Ser-264-α-P is due to both a perturbation of the active site structure and dynamics and to a repulsive interaction between the modified residue and PYR that leads to a reduced protein-ligand affinity.

In the cases of Ser-271-α and Ser-203-α, the picture emerging from our studies is less clear. In fact, the phosphorylation of Ser-271-α, seems to show only a small increase in protein flexibility that could however be the cause of its experimentally observed milder inhibitory effect^13,15,16^.

Regarding Ser-203-α-P, the effects of phosphorylation are completely different with respect to Ser-264-α-P and Ser-271-α-P. In this case, considering the debate about the strength of its influence on the PDC-E1 catalytic activity, we can hypothesize that this modification does not lead to enzyme inhibition or the inhibition is obtained by a different mechanism.

In this work, because of the lack of an experimentally solved atomistic structure of E1 in complex with the other subunits, as well as, the too large computational resources required to perform long enough atomistic simulations of the entire PDC, we chose to focus our investigation on a ‘minimal model’ able to recapitulate the most important features of the complex. Consequently, once more complete structural information and more potent computational resources become available, additional investigations should be carried out to gain a deeper understanding the effects of phosphorylation in the context of the entire system.

Finally, from a pharmacological point of view, the results of this work suggest that an allosteric modulation of the PDC-E1 is possible, and it could be a viable strategy to design new molecular tools aimed to modulate the enzymatic activity.

In particular, small organic molecules or peptides could be designed to mimic the effects of Ser-264-α-P. Alternatively, in the case of PDHA1 deficiency, the inhibitory effects of the two posttranslational modifications could be selectively blocked, hindering the interaction of PDK(s) and the PDC-E1 subunit near Ser-264-α for which the most potent inhibition was reported^15,16,21^.

## Computational Methods

### MD simulations

Molecular dynamics simulations were carried out starting from the structure of the E1-PDC human protein (PDB ID: 1NI4)^46^. The coordinates of PYR, not present in the 1NI4 crystal structure, were obtained by structural alignment with the structure of the E1-PDC protein from *Geobacillus stearothermophilus* (PDB ID: 3DV0)^11^. Furthermore, selenomethionine residues were mutated to methionine.

All molecular complexes were fully solvated in a TIP3P water box^47^ with a minimum distance of 10 Å from the protein surface. The charge neutrality of the systems was ensured by adding a proper number of counterions (Na^+^ ions).

The systems were described by Amber Force Field (ff14SB^48^ for the protein, phosaa10^49,50^ for the phosphorylated residues, GAFF^51^ for pyruvate and cofactors; the parameters for Na^+^ and Mg^2+^ were taken from references^52^ and^53^, respectively). Atomic charges for ThDP and PYR were computed with the AM1-bcc approach implemented in the ANTECHAMBER module of Amber16. Finally, MD simulations reported in this study were carried out with the GPU accelerated PMEMD code available in Amber16^54^.

To avoid distortions of the complexes, the systems were first minimized for 10.000 steps or until an energy gradient of 0.2 kcal mol^−1^Å^−1^ was reached. First, only water and counterions were left free to move, while the backbone atoms were restrained with a harmonic potential of 20 kcal mol^−1^Å^−1^. Then, the restraints were removed and the minimization procedure repeated. Finally, the temperature of the systems was increased to 298.5 K in 400 ps, and the pressure increased to 1 atm. During the MD simulations, the temperature and pressure were kept constant using the Langevin thermostat^55^ and Monte Carlo barostat^56^, respectively. Furthermore, the distance between the activated ThDP carbon and C2@PYR was restricted in the range between 3.3 Å and 3.7 Å by applying a flat-bottom harmonic (k = 80 kcal/mol/Å^2^) distance restraint.

### Analysis of the MD simulations

MD trajectories were analyzed using specific tools implemented in the programs VMD^57^, cpptraj^58^ and ALMOST^59^. The Lindemann coefficient^38^ was computed considering backbone atoms by the PCA suite program (http://mmb.pcb.ub.es/software/pcasuite/pcasuite.html). The Lindemann coefficient was first proposed to determine the solid or liquid character of an infinite system^60,61^ and subsequently applied to proteins by Karplus and coworkers^38^.

Protein tunnel analysis was done with the Caver 3.0 software^43^ on 1250 equally spaced snapshots (1 every 800 ps) extracted from the 1 μs MD simulations. All the water molecules and the pyruvate were removed from the structures. Furthermore, the origin of the tunnels was set to be the geometrical center of the following four atoms: (1) the nucleophile carbon of ThDP (Cn in Fig. 1), (2) Cα@Phe61α, (3) Cα@Tyr89α and (4) Cα@Phe86β (residue numbering refers to the human E1-PDC structure, PDB ID: 1NI4^46^). Finally, considering the size of PYR, the search of tunnels was performed using a probe with a radius of 1.5 Å.

In Caver analysis, each tunnel is described as an ensemble of spheres whose radius can fit the tunnel diameter. All identified tunnels were clustered by hierarchical average link using the pairwise distances between the centers of the spheres. The predicted tunnels were ranked according to the Carver score, designed to give priority to wide paths with a reasonable length^43^.

The affinity between the proteins and the substrate (PYR) was estimated using the MM-GBSA approach^62^.

MM-GBSA calculations were run on 2500 frames extracted (one every 400 ps) from the entire trajectories according to the following procedure: (1) water molecules and counterions were removed, (2) the same parameters used during MD simulations were assigned to both protein and ligand, and (3) the polar contribution to solvation energy was computed by the Onufriev, Bashford and Case model setting the dielectric constant to 1 for the solute and 80 for the solvent^63^. Per-residue decomposition (PRD) was used to estimate the contribution of single protein residues to the binding energy. All the calculations were run using the MMPBSA.py^62^ module available in AmberTools16.

The maximum information spanning tree (MIST) approach of Tidor and coworkers^64,65^ is a well-established method to estimate entropy from MD simulations^66–68^. The influence of the protein phosphorylations on conformational entropy were assessed using the implementation of the method in the recently released PDB2ENTROPY program^67^. To address this aim, 2500 snapshots were extracted from the MD simulations and processed using the following command line options ‘-mi -nt 16 -ne 40’.

The entropic contribution to the free energy caused by phosphorylation was defined as:

1 $$-{\rm{T}}{\rm{\Delta }}{\rm{S}}=-\,{\rm{T}}({{\rm{S}}}_{{\rm{phopshorylated}}}-{{\rm{S}}}_{{\rm{unphosphorylated}}})$$

How phosphorylation influences the hydrophobicity/hydrophilicity of PDC-E1 was assessed by computing the molecular hydrophobicity potential (MHP) using the PLATINUM webserver^69^. The difference between the sum of the MHP values over the protein solvent-accessible surface calculated for the phosphorylated and unphosphorylated protein (ΔMHP) was considered as measure of the effect of phosphorylation.

## Electronic supplementary material

Supplementary Information
