# Supplementary material for: How phosphorylation influences E1 subunit pyruvate dehydrogenase: A computational study
Source: Sci Rep. 2018 Oct 2;8:14683. doi: 10.1038/s41598-018-33048-z (PMC6168537; doi:10.1038/s41598-018-33048-z)
Supplement: Supplementary file 1 — Supplementary Information [file 41598_2018_33048_MOESM1_ESM.docx]

How phosphorylation influences E1 subunit pyruvate dehydrogenase: A computational study

Jacopo Sgrignani^1,2*^, JingJing Chen,^3^ Andrea Alimonti^3^ and Andrea Cavalli^1,2*^

^1^Institute for Research in Biomedicine (IRB), Università della Svizzera Italiana (USI), Via Vincenzo Vela 6, CH-6500, Bellinzona, Switzerland.

^2^ Swiss Institute of Bioinformatics, Lausanne, Switzerland.

^3^Institute of Research in Oncology (IOR), Università della Svizzera Italiana (USI), Via Vincenzo Vela 6, CH-6500, Bellinzona, Switzerland.

**SUPPORTING INFORMATION**

Corresponding author:

Andrea Cavalli

Institute for Research in Biomedicine

Via Vincenzo Vela 6

CH-6500 Bellinzona, Switzerland

Email: [andrea.cavalli@irb.usi.ch](mailto:andrea.cavalli@irb.usi.ch)

Jacopo Sgrignani

Institute for Research in Biomedicine

Via Vincenzo Vela 6

CH-6500 Bellinzona, Switzerland

Email: [jacopo.sgrignani@irb.usi.ch](mailto:jacopo.sgrignani@irb.usi.ch)

**Figure SI1.** Distances between the Cζ carbon of Arg273-α (red), Arg206-α (blue), Arg197−α (orange), and the phosphorus atom of Ser203-α over the entire Ser-203-α-P MD simulation.

**Figure SI2** Distances between the Cζ carbon of Lys39-β (orange), Arg273-α (blue), Arg275-α (red), Lys39−β (orange), and the phosphorus atom of Ser271-α over the Ser-271-α-P MD simulation.
